# Structural and functional properties of prefibrillar α-synuclein oligomers

**DOI:** 10.1038/srep24526

**Published:** 2016-04-14

**Authors:** Laura Pieri, Karine Madiona, Ronald Melki

**Affiliations:** 1 Paris-Saclay Institute of Neuroscience, Centre National de la Recherche Scientifique, Université Paris-Saclay, 91190 Gif-sur-Yvette, France

## Abstract

The deposition of fibrillar alpha-synuclein (α-syn) within inclusions (Lewy bodies and Lewy neurites) in neurons and glial cells is a hallmark of synucleinopathies. α-syn populates a variety of assemblies ranging from prefibrillar oligomeric species to fibrils whose specific contribution to neurodegeneration is still unclear. Here, we compare the specific structural and biological properties of distinct soluble prefibrillar α-syn oligomers formed either spontaneously or in the presence of dopamine and glutaraldehyde. We show that both on-fibrillar assembly pathway and distinct dopamine-mediated and glutaraldehyde-cross-linked α-syn oligomers are only slightly effective in perturbing cell membrane integrity and inducing cytotoxicity, while mature fibrils exhibit the highest toxicity. In contrast to low-molecular weight and unstable oligomers, large stable α-syn oligomers seed the aggregation of soluble α-syn within reporter cells although to a lesser extent than mature α-syn fibrils. These oligomers appear elongated in shape. Our findings suggest that α-syn oligomers represent a continuum of species ranging from unstable low molecular weight particles to mature fibrils via stable elongated oligomers composed of more than 15 α-syn monomers that possess seeding capacity.

Parkinson’s disease (PD), Multiple System Atrophy (MSA) and Dementia with Lewy Bodies (DLB) are devastating synucleinopathies^1^. The deposition of filamentous insoluble protein inclusions termed Lewy bodies and Lewy neurites whose main constituent is aggregated α-synuclein (α-syn) characterizes synucleinopathies^2^. α-syn is a 140 residues presynaptic protein that is believed to play an important role in the regulation of synaptic vesicle trafficking and release as well as neuronal survival^3^.

*In vitro*, under physiological conditions, α-syn assembles into fibrillar assemblies that are structurally similar to the amyloids found in Lewy bodies^4^. Metastable, prefibrillar oligomeric precursors form in the early stages of α-syn assembly into fibrils^5^,^6^. We have recently shown that mature α-syn fibrils are at least 1000-fold more toxic than their oligomeric precursors upon exposure of human neuroblastoma cells to equal particle concentrations of oligomeric and fibrillar α-syn^7^. α-syn fibrils are toxic because of their ability to bind plasma membrane components, affect the normal distribution of essential membrane proteins and plasma membrane integrity and trigger a cellular response^7^,^8^,^9^. Many recent evidences have also shown that fibrillar α-syn exogenously added/injected or released from neurons can spread in a prion-like manner by seeding the aggregation of soluble α-syn in the cytoplasm of recipient cells^10^,^11^,^12^,^13^,^14^,^15^,^16^,^17^,^18^,^19^. The above-mentioned evidences suggest a central role for fibrillar α-syn in the onset and progression of synucleinopathies.

Prefibrillar oligomeric α-syn has been proposed to contribute to neurodegeneration by perturbing cellular ion homeostasis^20^, by seeding the assembly of soluble α-syn into higher molecular weight aggregates^21^, and/or by imbalancing cellular proteostasis^22^,^23^. To date, an extremely large variety of early prefibrillar α-syn oligomeric species that differ in structure, molecular weight and morphology have been described^5^. This reflects soluble α-syn ability to populate multiple conformational states that yield distinct, often coexisting, assemblies. While some oligomeric species are on-fibrillization pathway, others are considered off-fibrillization pathway as they were shown to form amorphous, nonfibrillar assemblies^24^. In addition, both toxic and non-toxic α-syn oligomeric species^20^,^25^,^26^,^27^,^28^ have been reported. Finally, through its chameleon property^29^, α-syn has been shown to form distinct assemblies upon binding ligands, interacting with partners such as molecular chaperones or following posttranslational modifications. The neurotransmitter dopamine (DA) has been shown to promote the formation of stable, SDS-resistant α-syn oligomers both *in vitro* and in neurons^30^,^31^,^32^ by different mechanisms, including the formation of stable α-syn-DA-quinone adducts, methionine oxidation, or non-covalent interactions^33^. DA-mediated α-syn oligomerization is likely to be pathophysiologically relevant, possibly accounting for the selective vulnerability of dopaminergic neurons in PD. However, evidence has not yet been provided on whether DA-mediated α-syn oligomers are cytotoxic or not and their pathogenic role (if any) in synucleinopathies is unknown.

Overall, a plethora of studies have described on- or off-fibrillar assembly pathway, toxic or harmless, α-syn oligomeric species (reviewed in^34^) whose respective contributions to the onset and progression of synucleinopathies have not yet been elucidated. Such information is urgently needed to design therapeutic strategies able to specifically target pathogenic α-syn species thus slowing down disease progression.

Here, we compare the specific structural and biological properties of distinct α-syn oligomers i) spontaneously formed during α-syn fibrillization; ii) generated in the presence of DA; and iii) covalently cross-linked with glutaraldehyde (GA), one of the most widely used homobifunctional protein cross-linking reagents^35^, to prevent their dissociation and/or further organization into larger aggregates. We determined the molecular mass of each type of oligomer that we purified to homogeneity by analytical ultracentrifugation (AUC). This allowed us to compare the specific toxicity and seeding properties of distinct α-syn oligomeric species at identical particle concentrations using cultured neuroblastoma SH-SY5Y and Neuro2A cells. We show that spontaneously formed on-fibrillar assembly pathway and distinct DA-mediated and GA-cross-linked α-syn oligomers are only slightly effective in perturbing cell membrane integrity and inducing cytotoxicity, while mature α-syn fibrils highly affect plasma membrane permeability and exhibit the highest toxicity. Interestingly, large GA-cross-linked and DA-mediated α-syn oligomers seed the aggregation of endogenous soluble α-syn within reporter cells, although with much lower efficiency compared to fibrillar α-syn. This suggests that large soluble α-syn oligomers composed of more than 15–30 molecules arising spontaneously or in the presence of agents such as DA constitute a continuum with fibrillar α-syn and may contribute to some extent to the deleterious processes triggered by fibrillar α-syn.

## Results

### Structural characterization of purified on-fibrillar assembly pathway α-syn oligomers

**Figure 1:**
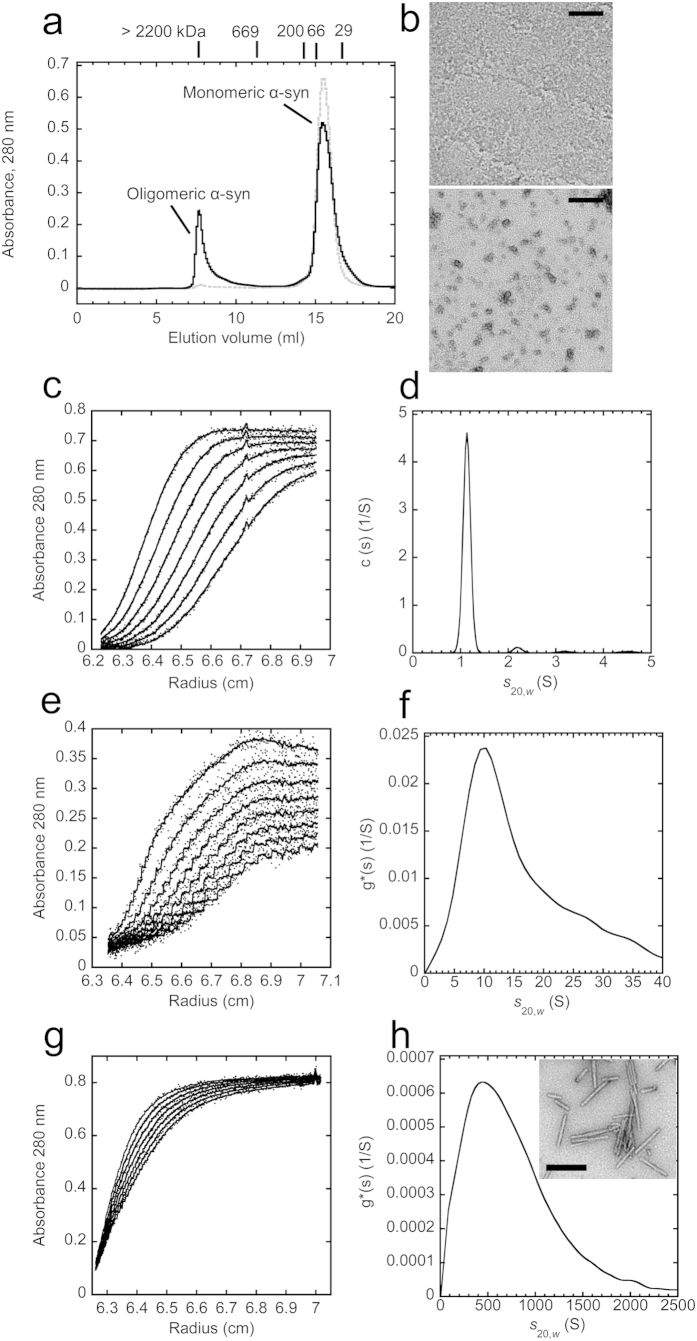
Production and characterization of on-fibrillar assembly pathway α-syn assemblies. (**a**) Isolation of on-fibrillar assembly pathway oligomeric α-syn by SEC. 500 μl of α-syn (800 μM monomer concentration) were loaded onto a Superose 6 (10/300) column and eluted at a flow rate of 0.5 ml/min in PBS diluted 10-fold. Elution was monitored by measuring absorbance at 280 nm. Grey dashed line, freshly thawed α-syn; Black solid line, α-syn incubated in assembly buffer for 7 days at 4 °C without shaking. The elution volumes of the standards Dextran blue (>2200 kDa); thyroglobulin (670 kDa); β-amylase (200 kDa); BSA (66 kDa); and carbonic anhydrase (29 kDa) are shown. Oligomeric α-syn eluted with an apparent molecular mass >2200 kDa. Monomeric α-syn eluted at ~54 kDa. (**b**) Negatively stained TEM images of purified monomeric (top panel) or oligomeric α-syn (bottom panel). Scale bars, 200 nm. (**c**) Sedimentation velocity data for monomeric α-syn (120 μM) at 50000 rpm and 15 °C in PBS diluted 10-fold. Shown are sedimentation boundaries obtained at intervals of 15 min. (**d**) Sedimentation coefficient continuous c(s) distribution of monomeric α-syn calculated from the sedimentation velocity data of panel (**c**) and corrected to *s*_20,*w*_. (**e**) Sedimentation velocity data for oligomeric α-syn (70 μM monomer concentration) at 25000 rpm and 15 °C in PBS diluted 10-fold. Shown are sedimentation boundaries obtained at intervals of 10 min. (**f**) Sedimentation coefficient g*(s) distribution of oligomeric α-syn calculated from the sedimentation velocity data of panel (**e**) and corrected to *s*_20,*w*_. (**g**) Sedimentation velocity data for fibrillar α-syn (20 μM monomer concentration) at 2000 rpm and 20 °C in assembly buffer. Shown are sedimentation boundaries obtained at intervals of 10 min. (**h**) Sedimentation coefficient g*(s) distribution of fibrillar α-syn calculated from the sedimentation velocity data of panel (**g**) and corrected to *s*_20,*w*_. Inset, negatively stained transmission electron micrograph of fibrillar α-syn. Scale bar, 200 nm.

We have previously assessed the functional properties of either mixed or purified monomeric and on-fibrillar assembly pathway α-syn oligomeric species^7^,^8^,^9^. Here we expand our comparison to distinct size-exclusion chromatography (SEC)-fractionated α-syn oligomers that form in the absence or the presence of DA and GA. Representative chromatograms of monomeric and oligomeric α-syn that form without any addition are shown in Fig. 1a. Freshly thawed α-syn eluted as a single peak with an apparent molecular weight of 54 kDa, consistent with the elution of natively unfolded monomeric α-syn^5^ (Fig. 1a, grey dashed line). When α-syn was incubated for 7 days at 4 °C in assembly buffer (50 mM Tris-HCl, pH 7.5, 150 mM KCl) without shaking, a peak emerging from the column with an apparent molecular weight >2200 kDa, representing 25% of total α-syn based on the peak area was observed beside the peak corresponding to monomeric α-syn (Fig. 1a, black solid line). This peak contained relatively homogeneous oligomeric α-syn that was absent in monomeric α-syn fraction as assessed by transmission electron microscopy (TEM) observations (Fig. 1b, lower and upper panel, respectively). Purified α-syn oligomers give rise to fibrillar assemblies (Supplementary Fig. S1) upon prolonged incubation (>30 days) under agitation at 37 °C at high concentration (250 μM monomeric α-syn). Therefore we will refer to them as on-fibrillar assembly pathway α-syn oligomers.

To assess the homogeneity of the preparation and determine with better precision their molecular weight we used AUC and sedimentation velocity measurements. For monomeric and oligomeric α-syn, no rapidly sedimenting material was detected while the rotor was accelerating to reach its operating speed, ruling out the presence of large aggregates. Fig. 1c,e,g show the sedimentation boundaries of monomeric, on-fibrillar assembly pathway oligomers and sonicated α-syn fibrils (see Methods for details), respectively. Analysis of the data for monomeric α-syn with the software Sedfit and the c(s) model^36^ yielded a single species with sedimentation coefficient (*s*_20,*w*_) of 1.13 S (*R*_H_ = 3.03; *f*/*f*_0_ = 1.88) (Fig. 1d). The sedimentation coefficient distributions of on-fibrillar assembly pathway α-syn oligomers and sonicated α-syn fibrils were obtained using the least-squares boundary modeling ls-g*(s)^37^. The ls-g*(s) distribution of on-fibrillar assembly pathway α-syn oligomers (Fig. 1f) is centered on a sedimentation coefficient of 9.9 S corresponding to particles with an average molecular mass of ~450 kDa, i.e. consisting of ~30 monomers (450 kDa/14.5 KDa).

The ls-g*(s) distribution for fibrillar α-syn (Fig. 1h) revealed the presence of particles with sedimentation coefficients ranging from 150 to 1500 S, centered at ~435 S, corresponding to particles of ~120,000 kDa, i.e. made of ~8300 *α*-syn monomeric units in agreement with previous measurements^7^. These measurements allowed us to determine the average particle concentration of purified on-fibrillar assembly pathway α-syn oligomers corresponding to a given equivalent monomer concentration. At a working concentration of 10 μM, the particle concentration of α-syn oligomers is 10 μM/30 = 0.33 μM, while the particle concentration of α*-*syn fibrils is 10 μM/8300 = 0.001 μM.

### Functional characterization of purified on-fibrillar assembly pathway α-syn oligomers

**Figure 2:**
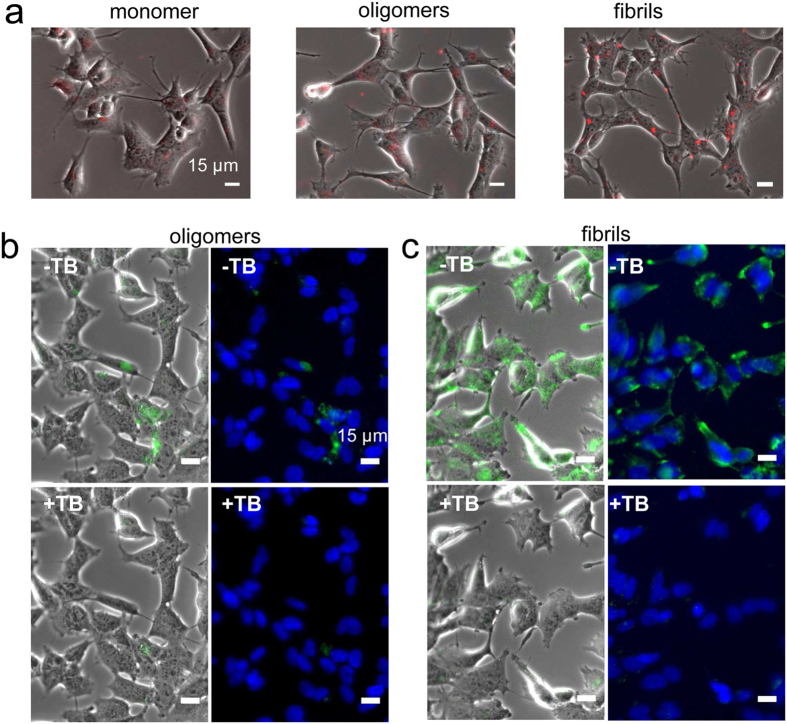
Interaction of α-syn assemblies with cell membranes. (**a**) Binding of ATTO550-labeled α-syn assemblies (red) to SH-SY5Y cells, imaged by epifluorescence and phase contrast microscopy. Left panel, monomeric α-syn (1 μM); Middle panel, oligomeric α-syn (30 nM, i.e. 1 μM monomeric α-syn); Right panel, fibrillar α-syn (0.1 nM, i.e. 1 μM monomeric α-syn). Scale bars, 15 μm. (**b**,**c**) Localization of ATTO488-labeled α-syn assemblies (green) on the plasma membranes of SH-SY5Y cells. Cells were exposed 15 min to 30 nM oligomeric (**b**) or 0.1 nM fibrillar α-syn (**c**) and imaged before (**b**,**c**, top panels, -TB) and after addition of the fluorescence quencher Trypan blue (**b**,**c**, bottom panels, +TB). Phase contrast imaging (**b**,**c**, left panels) and cell nuclei counterstaining (blue) with Hoescht 33258 (Invitrogen) (**b**,**c**, right panels) are also shown. Scale bars, 15 μm.

**Figure 3:**
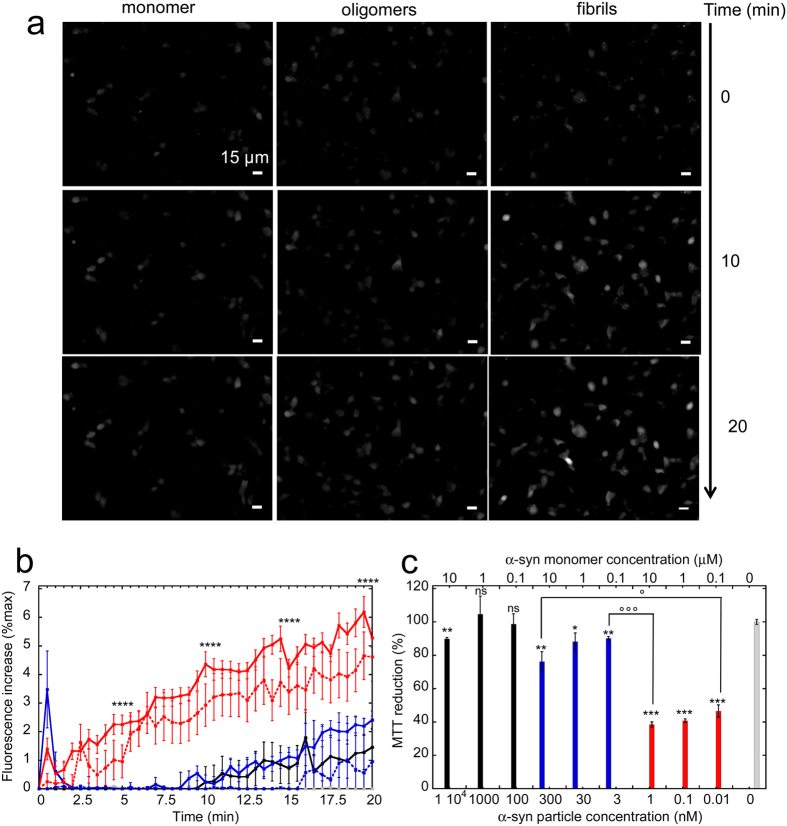
Intracellular free Ca^2+^ variation induced by α-syn assemblies and cellular toxicity. (**a**) Epifluorescence microscopy imaging of intracellular Ca^2+^ variation observed at 3 different incubation stages of Fluo-4-AM-loaded SH-SY5Y cells exposed to α-syn assemblies. Cells were imaged every 30 s for 20 min. Left panels, control cells exposed to monomeric α-syn (10 μM). Middle panels, oligomeric α-syn (300 nM, i.e. 10 μM monomeric α-syn); Right panels, fibrillar α-syn (1 nM, i.e. 10 μM monomeric α-syn). Scale bars, 15 μm. (**b**) Kinetics of intracellular Ca^2+^ variation induced by α-syn assemblies. Black curve, 10 μM monomeric α-syn. Blue curves, 300 nM (solid line) or 1 nM (dashed line) oligomeric α-syn; red curves, 1 nM (solid line) or 0.2 nM (dashed line) fibrillar α-syn. Grey curve, control cells treated with PBS. The fluorescence increase at each time point is expressed as a fraction of the maximum fluorescence measured upon addition of 10 μM ionomycin to the cells at the end of each experiment. Data are mean ± SE of the fluorescence increase measured in at least 100 different cells from 3 independent experiments. Fibrils 1 nM vs oligomers 300 nM, ****P < 0.0001 (two-sample, two-tailed independent Student’s t-test). (**c**) Viability of SH-SY5Y cells exposed 24 h to different particle concentrations of α-syn assemblies, measured by MTT assay. The initial monomeric protein concentration values corresponding to each particle concentration are indicated on the top x-axis. Black bars, monomeric α-syn; blue bars, oligomeric α-syn; red bars, fibrillar α-syn. Grey bar, PBS. MTT reduction is expressed as percentage relative to control cells treated with identical volumes of PBS. Data are mean ± SE (n = 6). Versus control cells, *P < 0.05; **P < 0.01; ***P < 0.001 (two-sample, two-tailed independent Student’s t-test). Oligomers vs fibrils, °P < 0.05; °°°P < 0.001 (two-sample, two-tailed independent Student’s t-test).

Next, we documented the functional properties of purified on-fibrillar assembly pathway α-syn oligomers and compared them to those of monomeric and fibrillar α-syn at similar particle concentrations. First, we checked the ability of α-syn oligomers to bind and permeabilize the cell membranes. To this aim, we exposed human neuroblastoma SH-SY5Y cells to fluorescently labeled monomeric, oligomeric or fibrillar α-syn and imaged the binding of assemblies to the cells by epifluorescence microscopy. In agreement with our previous observations^7^,^8^,^13^, fibrillar α-syn efficiently bound the cell surfaces after 15 min incubation (Fig. 2a, right panel). Purified on-fibrillar assembly pathway α-syn oligomers were found to interact with cells to a lesser extent (Fig. 2a, middle panel), whereas no or marginal binding was observed for monomeric α-syn (Fig. 2a, left panel). To determine whether α-syn assemblies were bound to the plasma membrane or within the cytosol after take up, we imaged the cells exposed for 15 min to ATTO488-labeled α-syn oligomers and fibrils before and after the addition of 0.1% Trypan blue. The latter quenches the fluorescence of assemblies that are bound to the extracellular side of the plasma membranes and not yet within the cells. Trypan blue efficiently quenched the fluorescence of on-fibrillar assembly pathway α-syn oligomers (Fig. 2b) and fibrils (Fig. 2c), indicating that most, if not all, α-syn assemblies are still extracellularly bound to the plasma membrane at this stage of incubation with the cells. We next investigated the consequences of α-syn binding to cell membranes. Membrane disruption and Ca^2+^ dysregulation are early events in the cascade triggered by toxic protein assemblies eventually leading to cell death^7^,^20^. We therefore measured the variations in intracellular Ca^2+^ levels in cells loaded with the fluorescent indicator Fluo-4-acetoxymethyl ester (Fluo-4-AM) and exposed to increasing concentrations of monomeric, oligomeric or fibrillar α-syn particles. 1 nM (Fig. 3a, right panels and Fig. 3b, red curve, solid line) and 0.2 nM α-syn fibrils (Fig. 3b, red curve, dashed line) induced a progressive and significant increase of intracellular Ca^2+^ levels, as revealed by the rise of Fluo-4 fluorescence in exposed SH-SY5Y cells. In contrast, only a modest Ca^2+^ increase was observed in cells exposed to 300 nM on-fibrillar assembly pathway oligomeric α-syn (Fig. 3a, middle panels and Fig. 3b, blue curve, solid line) or 10 μM monomeric α-syn (Fig. 3a, left panels and Fig. 3b, black curve, solid line). Interestingly, an early burst in Fluo-4 fluorescence was recorded during the first 60 sec of cell exposure to 300 nM on-fibrillar assembly pathway oligomeric α-syn. Such burst, however, was followed by a rapid decline of fluorescence levels (Fig. 3b, blue curve, solid line) and was not observable at lower concentrations (1 nM) of oligomeric α-syn (Fig. 3b, blue curve, dashed line).

We next compared the cytotoxicity of α-syn monomers, oligomers and fibrils at increasing particle concentrations using SH-SY5Y cells and the 3-(4,5-dimethylthiazol-2-yl)-2, 5-diphenyltetrazolium bromide (MTT) reduction inhibition assay, a widely used indicator to assess cell viability (see Methods). α-syn fibrils revealed to be highly toxic to cells at all the concentrations we tested, spanning 1 to 0.01 nM (53.4 ± 4% inhibition of MTT reduction at 0.01 nM, i.e. 0.1 μM equivalent monomer concentration) whereas α-syn oligomers only slightly impaired cell viability (23.9 ± 6% inhibition of MTT reduction at 300 nM, i.e. 10 μM initial monomer concentration) (Fig. 3c). Monomeric α-syn exhibited very low toxicity only at the highest tested concentration (10.2 ± 1% inhibition of MTT reduction at 10 μM) (Fig. 3c).

### Generation and structural characterization of stable α-syn oligomers

By definition, on-fibrillar assembly pathway α-syn oligomers are metastable intermediates that rapidly grow into larger aggregates. We therefore checked whether and to what extent purified α-syn oligomers remain stable over time.

After purification and further incubation in PBS at a particle concentration of 1 μM (equivalent to 30 μM monomeric α-syn) for up to 2 days at 37 °C under agitation, α-syn oligomers retained the same morphology upon TEM analysis (Supplementary Fig. S1) and eluted at the same volume when re-injected onto the Superose 6 column (not shown). Larger assemblies, including short chains, rings, as well as short fibrils, along with the early oligomeric precursors were apparent upon TEM analysis when the oligomers were incubated in PBS at higher concentrations (8 μM particle concentration i.e. 250 μM monomeric α-syn) at 37 °C under agitation for 7 days (Supplementary Fig. S1). Increasing amounts of fibrils and a concomitant decrease in the amount of oligomeric species were observed upon longer incubation times (Supplementary Fig. S1). These observations indicate that on-fibrillar assembly pathway α-syn oligomers further evolve with time when incubated at high concentrations. Next, we generated stable populations of α-syn oligomers i) by favoring oligomerization in the presence of DA and ii) by chemically cross-linking on-fibrillar assembly pathway α-syn oligomers with GA, as described in the Methods section.

**Figure 4:**
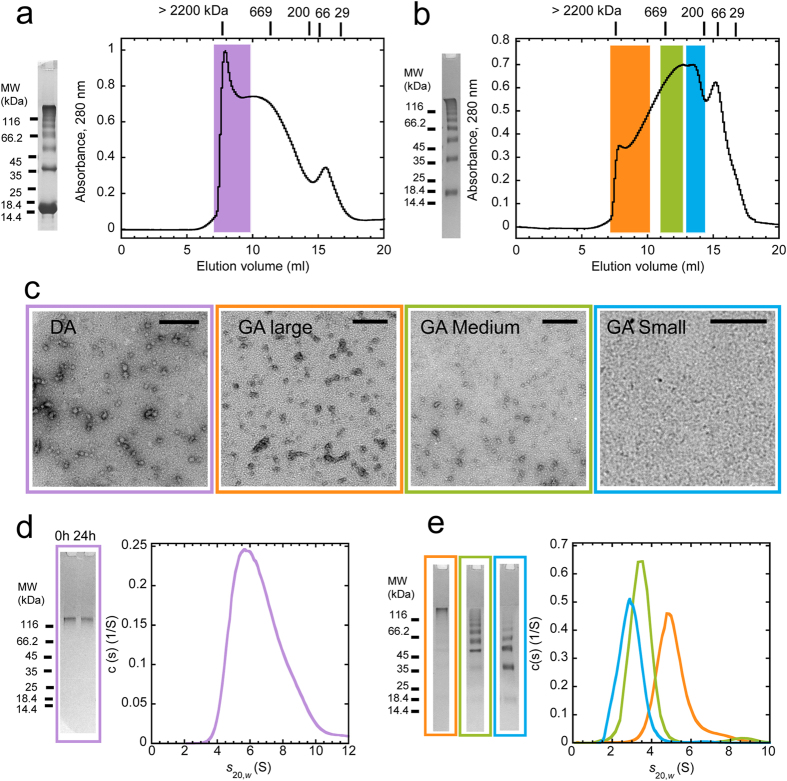
Production and characterization of SDS-stable DA-mediated or GA-cross-linked α-syn oligomers. (**a**,**b**) Isolation of DA-mediated (**a**) or GA-cross-linked (**b**) α-syn oligomeric species by SEC. Coomassie blue-stained 10% Tris-Tricine-SDS-PAGE of SDS-resistant oligomeric populations obtained in the presence of DA (**a**) or GA (**b**) are also shown. α-syn (500 μl, 600 μM) incubated in the presence of DA or GA (see Methods for details) was loaded onto a Superose 6 (10/300) column and eluted at a flow rate of 0.5 ml/min in PBS diluted 10-fold. Elution was monitored by measuring absorbance at 280 nm. The elution positions of the standards Dextran blue (>2200 kDa); thyroglobulin (670 kDa); β-amylase (200 kDa); BSA (66 kDa); and carbonic anhydrase (29 kDa) are indicated. Colored areas in panels (**a**,**b**) indicate the four different populations of DA-mediated and GA-cross-linked α-syn oligomers we isolated and characterized. Purple area, large DA-mediated oligomers. Orange area, large; green area, medium; cyan area, small GA-cross-linked α-syn oligomers (also see Supplementary Fig. S2). (**c**) Negatively stained TEM images of purified DA-mediated (purple frame); large (orange frame), medium (green frame), and small (cyan frame) GA-cross-linked α-syn oligomers. Scale bars, 200 nm. (**d**) Sedimentation coefficient continuous c(s) distributions of isolated DA-induced α-syn oligomers, obtained by sedimentation velocity measurements performed at 25000 rpm and 15 °C, and corrected to *s*_20,*w*_. Coomassie blue-stained 10% Tris-Tricine-SDS-PAGE profiles of isolated DA-mediated α-syn oligomers before and after 24 h incubation in serum free Dulbecco’s modified Eagle’s Medium/Ham’s nutrient mixture F-12 (DMEM/F12) cell culture medium are also shown. (**e**) Sedimentation coefficient continuous c(s) distributions of purified large (orange curve), medium (green curve) and small GA-cross-linked α-syn oligomers (cyan curve), obtained by sedimentation velocity measurements performed at 25000 rpm and 15 °C, and corrected to *s*_20,*w*_. Coomassie blue-stained 10% Tris-Tricine-SDS-PAGE of purified large (orange frame), medium (green frame) and small GA-cross-linked α-syn oligomers (cyan frame) are also shown.

DA-mediated α-syn oligomers constitute a range of SDS-resistant species with apparent molecular weights ranging from over 2200 to 200 kDa as determined by SEC (Fig. 4a). Monomeric α-syn eluted from SEC with an apparent molecular weight of ~55 KDa (as in Fig. 1a). The oligomeric species that was most represented had an apparent molecular weight of over 2200 kDa. GA-cross-linked α-syn oligomers are also a heterogeneous set of SDS-resistant oligomeric species (Fig. 4b). In contrast to DA-mediated α-syn oligomers, the most abundant GA-cross-linked α-syn oligomeric species had an apparent molecular weight ranging from 700 to 220 kDa (Fig. 4b). The DA-mediated oligomeric species that was most represented (with an apparent molecular weight of over 2200 kDa, highlighted in purple in Fig. 4a) and three distinct pools of GA-cross-linked α-syn oligomers highlighted in orange, green and cyan in Fig. 4b and named (large, medium and small GA-oligomers, respectively) based on SDS-PAGE analysis of GA-cross-linked α-syn SEC fractions (Supplementary Fig. S2) were selected for further analysis. The morphology of DA-mediated and differently sized GA-cross-linked α-syn oligomers was first assessed by TEM (Fig. 4c). Next, we performed sedimentation velocity measurements to determine with greater precision the molecular weights of the different DA-mediated and GA-cross-linked α-syn oligomers fractions. The DA-α-syn oligomers corresponding to the main peak fraction highlighted in purple in Fig. 4a had a sedimentation coefficient c(s) distribution centered on ~5.7 S (f/f0 = 2.8) (Fig. 4d). This size corresponds to particles with an average molecular weight of ~300 kDa, i.e. composed of ~20 monomers, consistent with the migration profile on SDS-PAGE (Fig. 4d). Notably, when DA-mediated α-syn oligomers were incubated for 24 h at 37 °C in PBS or serum-free culture medium, their SDS-PAGE profile remained unchanged, indicating that these species retain an SDS-stable oligomeric structure over time (Fig. 4d). To further ascertain the stability of DA-mediated α-syn oligomers, we used Semi-Denaturing Detergent-Agarose Gel Electrophoresis (SDD-AGE) and TEM. Supplementary Fig. S3 shows that the SDD-AGE migration of DA-mediated α-syn oligomers incubated 24 h in the cell culture medium of SH-SY5Y cells is identical to that of freshly purified oligomers, but different from that of monomeric and fibrillar α-syn. Moreover, DA-mediated α-syn oligomers retain their initial morphology when incubated for up to 7 days under the same conditions, as assessed by TEM (Supplementary Fig. S3).

The migration on SDS-PAGE and the sedimentation coefficient c(s) distributions of large, medium and small GA-cross-linked α-syn oligomers are shown in Fig. 4e. The sedimentation coefficient distribution of large GA-cross-linked α-syn oligomers is centered on 4.82 S (f/f0 = 2.8) corresponding to a molecular weight of 228 kDa consistent with a particle made of ~16 monomers (Fig. 4e, orange curve). Medium GA-cross-linked α-syn oligomers have an average sedimentation coefficient of 3.36 S (f/f0 = 2.1) corresponding to an average molecular weight of 88 kDa i.e. ~6 monomers (Fig. 4e, green curve). Small GA-cross-linked α-syn oligomers have an average sedimentation coefficient of 2.89 S (f/f0 = 2.0) corresponding to particles with an average molecular weight of 64 kDa i.e. ~4 monomers (Fig. 4e, cyan curve). Notably, the high (>1.25) frictional ratios (f/f0) the c(s) analysis yields suggest that α-syn oligomers are elongated/asymmetric species, consistent with their high apparent molecular mass determined by SEC. Determination of the number of monomeric α-syn within each type of oligomer we generated allowed us to calculate the particle concentration at any monomeric α-syn concentration.

### Functional characterization of stable α-syn oligomers

**Figure 5:**
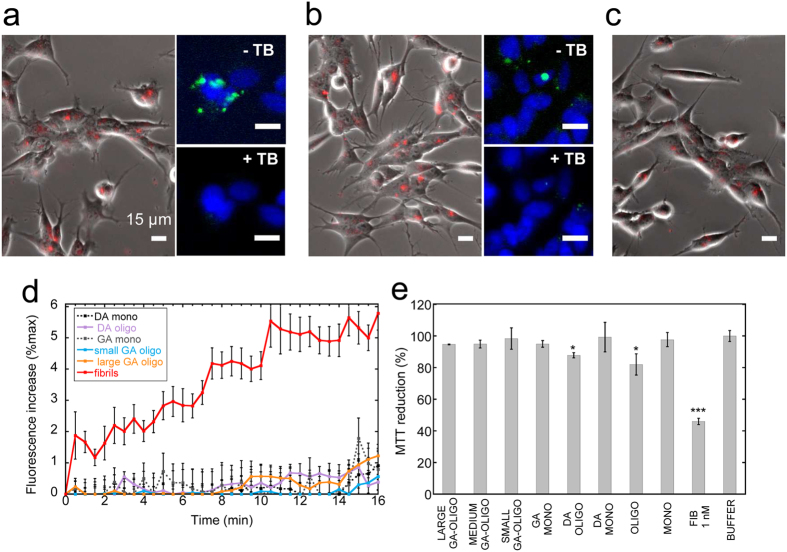
Interaction of DA-mediated or GA-cross-linked α-syn oligomers with cell membranes, intracellular free Ca^2+^ and toxicity measurements. (**a**–**c**) binding of ATTO550-labeled DA-mediated or GA-cross-linked α-syn oligomers (red) to the surfaces of human neuroblastoma SH-SY5Y cells, imaged by epifluorescence and phase contrast microscopy. (**a**) DA-mediated α-syn oligomers (50 nM, equivalent to 1 μM monomeric α-syn); (**b**) large GA-cross-linked α-syn oligomers (62.5 nM, equivalent to 1 μM monomeric α-syn); (**c**) small GA-cross-linked α-syn oligomers (250 nM, equivalent to 1 μM monomeric α-syn). Small panels on the right in (**a**,**b**) show ATTO488-labeled DA-mediated α-syn oligomers (**a**) and large GA-cross-linked α-syn oligomers (**b**) (green) imaged before (top, -TB) and after (bottom, +TB) the addition of Trypan blue to quench the fluorescence of plasma membrane-bound oligomeric species. Cell nuclei were counterstained with Hoescht 33258 (blue). Scale bars, 15 μm. (**d**) kinetics of intracellular free Ca^2+^ variation monitored by epifluorescence microscopy imaging of SH-SY5Y cells loaded with Fluo-4-AM and exposed to DA-mediated or GA-cross-linked α-syn oligomers. Cells were imaged every 30 s for 20 min. Black dotted curve, 10 μM monomeric α-syn coming from the SEC separation of α-syn incubated in the presence of DA (see Fig. 4a); purple curve, 500 nM DA-mediated α-syn oligomers; grey dotted curve, 10 μM monomeric α-syn coming from the SEC separation of α-syn incubated in the presence of GA (see Fig. 4b); orange curve, 625 nM large GA-cross-linked α-syn oligomers; cyan curve, 2.5 μM small GA-cross-linked α-syn oligomers; red curve, 1 nM fibrillar α-syn. The fluorescence increase at each time point is expressed as a fraction of the maximum fluorescence measured upon addition of 10 μM ionomycin to the cells at the end of each experiment. Data are mean ± SE of the fluorescence increase measured in at least 100 different cells from 3 independent experiments. (**e**) Viability of SH-SY5Y cells exposed for 24 h to monomeric and oligomeric DA-mediated or GA-cross-linked α-syn (300 nM) or to fibrillar α-syn (1 nM), measured by MTT assay. MTT reduction is expressed as percentage relative to control cells treated with identical volumes of PBS. Data are mean ± SE (n = 6). Versus control cells, *P < 0.05; ***P < 0.001 (two-sample, two-tailed independent Student’s t-test).

We next assessed the ability of stable α-syn oligomers to bind/permeabilize cell membranes. Both DA-mediated and GA-cross-linked oligomers bound the surfaces of SH-SY5Y cells after 15 min incubation (Fig. 5a–c). The ability of Trypan blue to quench the fluorescence of cell-bound oligomers (small panels in Fig. 5a,b) indicates that, as for on-fibrillar assembly pathway α-syn oligomers and fibrils (Fig. 2b,c), stable α-syn oligomers are plasma membrane-bound and not yet within the cells at this stage of incubation. Similarly to on-fibrillar assembly pathway α-syn oligomers (Fig. 3a,b), none of the stable oligomeric α-syn species altered intracellular Ca^2+^ levels of the cells (Fig. 5d). In agreement with this, limited cytotoxicity was measured. Fig. 5e shows that 30 nM DA-mediated α-syn oligomers impaired cell viability to the same extent as on-fibrillar assembly pathway α-syn oligomers (~12% as compared to 18% inhibition of MTT reduction). Large, medium and small GA-cross-linked oligomers at identical concentrations exhibited no toxicity (Fig. 5e). These observations indicate that on-fibrillar assembly pathway α-syn oligomers and stable α-syn oligomers toxicity is negligible when compared to that of fibrillar α-syn.

### Seeding properties of α-syn oligomers

We, and others, have recently shown that exogenous α-syn fibrils are internalized by cultured cells and *in vivo* in animal models and amplify by seeding the aggregation of soluble endogenous α-syn^12^,^13^,^14^,^15^,^16^,^17^,^18^,^19^. A seeding activity *in vitro* and in cultured cells has also been described for oligomeric α-syn^21^,^38^. We therefore compared the seeding propensities of the distinct oligomeric assemblies we generated to that of fibrillar α-syn.

Murine neuroblastoma Neuro2A cells stably expressing α-syn fused to mCherry fluorescent protein (ChFP–α-syn)^13^ were exposed to increasing concentrations of the different oligomeric α-syn assemblies labeled with ATTO488. The pattern of endogenous cytoplasmic ChFP-α-syn and the colocalization of aggregated ChFP-α-syn with ATTO-488-fluorescence were assessed by epifluorescence microscopy.

**Figure 6:**
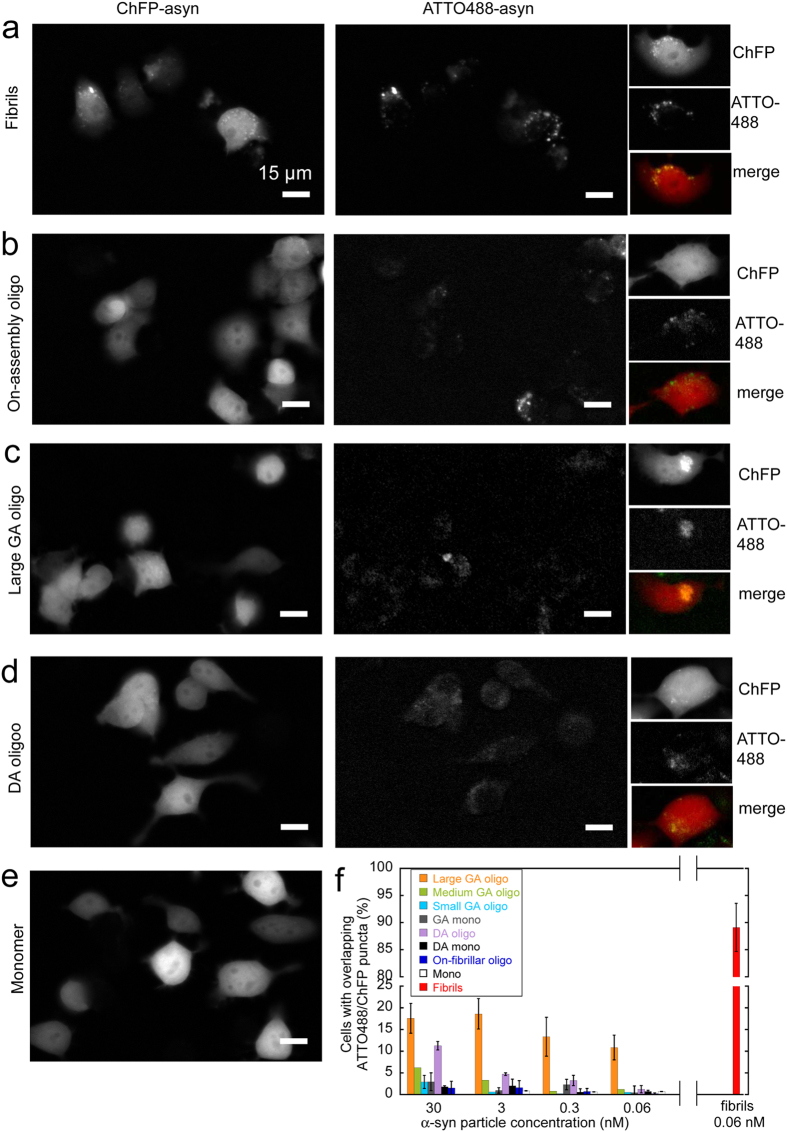
Seeding propensity of α-syn oligomers assessed by epifluorescence microscopy imaging. (**a**–**e**) Epifluorescence microscopy imaging of Neuro2A cells stably expressing ChFP-α-syn exposed 24 h to 1 nM α-syn fibrils (**a**); 30 nM on-fibrillar assembly pathway α-syn oligomers (**b**); 30 nM large GA-cross-linked α-syn oligomers (**c**); 30 nM DA-mediated α-syn oligomers (**d**); or 0.5 μM monomeric α-syn (**e**). α-syn assemblies were labeled with ATTO488. Left panels, ChFP-α-syn red fluorescence; middle panels, ATTO488-α-syn green fluorescence; Right panels show ChFP and ATTO488 fluorescence as well as merged images for representative individual cells. Scale bars, 15 μm. (**f**) Proportion of cells with exogenous ATTO-488-labeled α-syn assemblies overlapping with endogenous cytoplasmic ChFP-α-syn puncta after 24 h exposure to different concentrations of ATTO-488-labeled α-syn assemblies. Values are mean ± SE, obtained by randomly counting at least 500 cells in 7–10 fields from 3 independent experiments.

Upon exposure of cells to 0.06 nM ATTO-488-labeled α-syn fibrils (equivalent to 0.5 μM monomeric α-syn) for 24 h, the normally even ChFP-α-syn fluorescence (shown in Fig. 6e, control cells exposed to 0.5 μM monomeric α-syn) redistributed into distinct puncta that overlapped with exogenous ATTO-488-labeled α-syn fibrils (Fig. 6a). The high proportion of cells with overlapping ChFP and ATTO-488 puncta (89 ± 7% upon cell exposure to 0.06 nM ATTO-488-labeled α-syn fibrils, Fig. 6f) indicates that α-syn fibrils seed with high efficiency the aggregation of soluble cytoplasmic ChFP-α-syn. In contrast, ATTO-488-labeled on-fibrillar assembly pathway α-syn oligomers (up to 30 nM, equivalent to 1 μM monomeric α-syn), although clearly visible within exposed cells (Fig. 6b), did not induce the formation of ChFP-α-syn foci (Fig. 6f). A significant proportion of cells exposed to large GA-cross-linked α-syn and DA-mediated oligomers (0.06–30 nM, equivalent to 1–600 nM monomeric α-syn) displayed overlapping ChFP and ATTO488 puncta (Fig. 6c,d,f). In contrast, medium and small GA-cross-linked α-syn oligomers, and monomeric α-syn exposed or not to GA or DA (Fig. 6e) did not induce ChFP-α-syn aggregation at the highest concentration we used (Fig. 6f). We conclude from these observations that on-fibrillar assembly pathway, medium and small GA-cross-linked α-syn oligomers do not seed the aggregation of endogenous α-syn while large GA-cross-linked and DA-mediated α-syn oligomers do.

**Figure 7:**
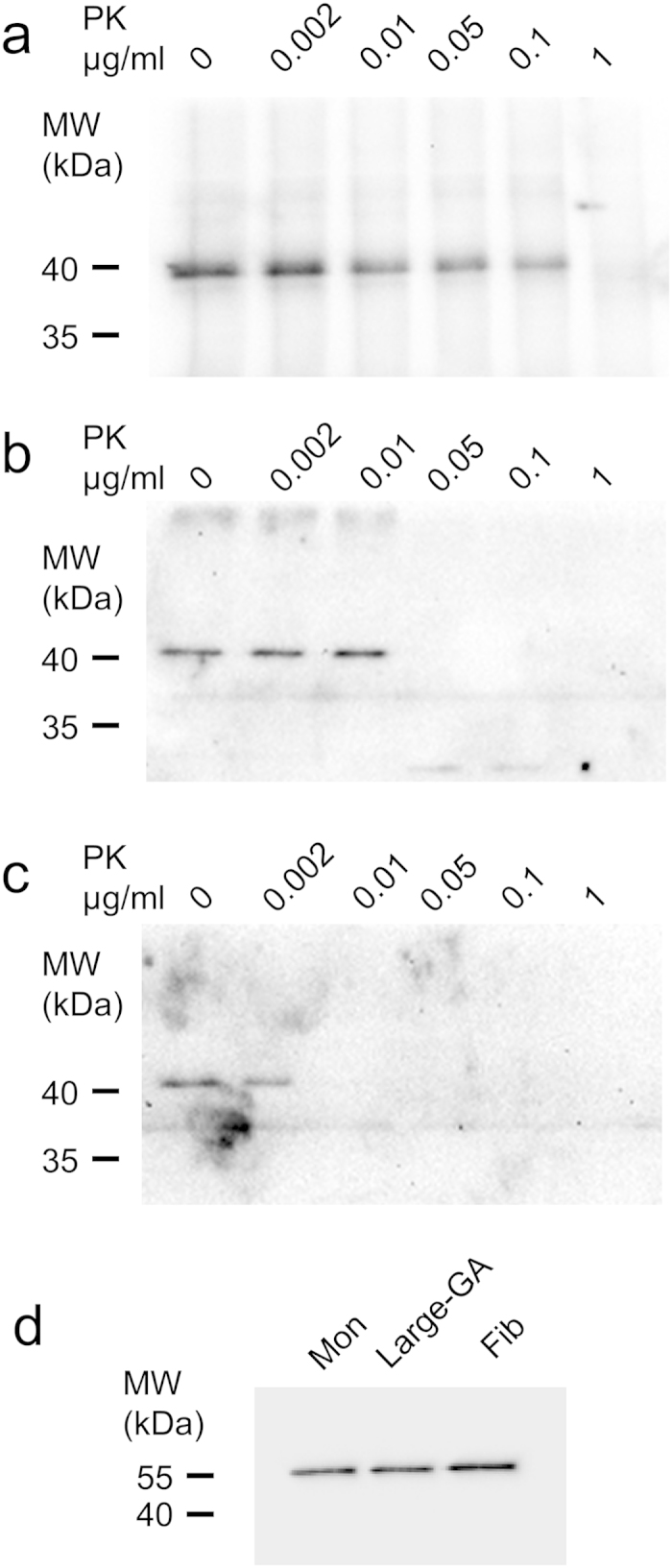
Seeded aggregation of reporter ChFP-α-syn by exogenous α-syn assemblies assessed by increased resistance to proteolysis. Western blot analysis of the ChFP-α-syn resistance to proteinase K in lysates from Neuro2A cells exposed for 24 h to 0.3 nM α-syn fibrils, equivalent to 2.5 μM monomeric α-syn (**a**), 300 nM large GA-cross-linked α-syn oligomers, equivalent to 5 μM monomeric α-syn (**b**), or 5 μM monomeric α-syn (**c**). The lysates (40 μl corresponding to ~80000 cells), were incubated in the presence of the indicated concentrations of proteinase K for 20 min at 37 °C. The proteolytic reactions were stopped by addition of 1 mM PMSF and immediate denaturation in Laemmli buffer for 5 min at 95 °C. The samples were analyzed on 12% Tris-Glycine SDS-PAGE. ChFP–α-syn (**a**–**c**) was probed with mouse monoclonal anti-α-syn antibody (BD Biosciences Cat #610787). The immunoreactivity of α-tubulin (mouse monoclonal antibody DM1A, Abcam Cat #ab7291) in the initial lysate was used as a loading control (**d**). ChFP-α-syn assemblies seeded by α-syn fibrils resisted 0.1 μg/ml proteinase K (**a**). ChFP-α-syn from cells exposed to monomeric α-syn was fully degraded by 0.01 μg/ml proteinase K (**c**). ChFP-α-syn originating from cells exposed to large GA-cross-linked oligomers resisted 0.01 μg/ml and was fully degraded by 0.05 μg/ml proteinase K (**b**).

To strengthen this conclusion and demonstrate that large and stable α-syn oligomers recruit soluble ChFP-α-syn and convert it into insoluble aggregates in a manner similar to fibrils, we assessed the resistance to proteolysis of ChFP-α-syn in lysates from cells exposed for 24 h to large GA-cross-linked α-syn oligomers. Control lysates from cells exposed for 24 h to monomeric or fibrillar α-syn were also prepared. Cell lysates were incubated in the presence of increasing concentrations of proteinase K and the proteolytic degradation of ChFP-α-syn seeded or not by exogenous α-syn assemblies was assessed by western blot analysis. As expected and in agreement with previous reports^13^, ChFP-α-syn originating from cells exposed to α-syn fibrils exhibited a significant resistance to proteolysis (Fig. 7a). ChFP-α-syn from cells exposed to monomeric α-syn was fully degraded with as little as 0.01 μg/ml proteinase K (Fig. 7c). ChFP-α-syn originating from cells exposed to large GA-cross-linked oligomers exhibited an intermediate resistance to proteolysis, with complete degradation by 0.05 μg/ml proteinase K (Fig. 7b). This observation is in agreement with the seeding propensity we report in Fig. 6.

## Discussion

As Lewy Bodies are intracellular, α-syn assemblies have long been considered to exert their toxicity intracellularly. However, the observations that α-syn assemblies can be released into the extracellular medium and traffic between neurons^11^,^39^,^40^ and that α-syn assemblies are detectable in body fluids such as serum and the cerebrospinal fluid of patients developing synucleinopathies^41^,^42^ support the growing idea that extracellular α-syn assemblies play a role in synucleinopathies^43^. For more than a decade, prefibrillar oligomers formed at early stages of α-syn assembly into fibrils have been proposed to be involved in neurodegeneration^44^,^45^,^46^,^47^. This is based on the ability of oligomers to interact with lipid membranes leading to their destabilization^20^,^48^,^49^ and on the increased levels of oligomeric α-syn in the cerebrospinal fluid of patients affected by synucleinopathies^42^. However, direct evidence that oligomeric α-syn is responsible for neurodegeneration in PD is lacking and the pathogenic role of α-syn oligomers is unclear in our opinion as the exact nature of α-syn assemblies that are termed oligomers in the abovementioned studies is ill-defined. In addition, while artificial α-syn variants that specifically form oligomers rather than fibrils have been shown to be associated with increased toxicity when expressed in invertebrate^50^ and rat using lentiviral vectors^46^, recent reports indicate that α-syn variants that are more prone to form fibrils induce a more significant loss of dopaminergic neurons in the rat substantia nigra^51^. Finally, we and others have recently shown that fibrillar α-syn assemblies are significantly more toxic than their oligomeric precursors^7^,^8^,^9^,^19^,^51^,^52^,^53^,^54^,^55^.

α-syn can form a large variety of oligomeric species that differ in structure, size and morphology because of its high structural plasticity^29^,^34^. Hence, both toxic^20^,^22^,^23^,^25^ and nontoxic^7^,^8^,^9^,^26^,^27^,^28^ α-syn oligomeric species have been reported. In a manner similar to fibrils, α-syn oligomers are taken up by neurons and transported through interconnected regions of the peripheral and central nervous system^12^,^14^,^15^,^16^,^17^,^18^,^19^ reflecting the spatiotemporal pattern of Lewy pathology propagation characteristic of PD^56^. However, it is still unclear whether the distinct oligomeric α-syn species that have been described so far contribute to the prion-like spreading of the pathology by seeding the aggregation of soluble α-syn in recipient neurons.

We compared here the structural and biological properties of distinct α-syn oligomers (e.g. on-fibrillar assembly pathway, DA-mediated and GA-cross-linked) we purified. On-fibrillar assembly pathway α-syn oligomers are constituted on average by ~30 α-syn molecules, in agreement with data obtained under slightly different conditions^49^. They display weak membrane permeabilization efficiency compared to fibrils. Interestingly however we observed consistently an early burst of Ca^2+^ concentration within cells within 30 s after exposure to α-syn oligomers but not monomers (Fig. 3b). This may be related to the clustering of oligomeric α-syn within the cell membrane we recently reported^8^. While cells exposed to 300 nM on-fibrillar assembly pathway α-syn oligomers (i.e. 10 μM monomeric α-syn) were able to restore the basal Ca^2+^ levels within seconds, exposure of the same cells to 300-to-1500-fold lower concentrations of fibrillar α-syn (i.e. identical to 6 fold lower concentrations of monomeric α-syn) led to a progressive and significant increase of intracellular Ca^2+^ levels that was not reversed over 20 minutes. This observation accounts for the higher specific toxicity of α-syn fibrils compared to oligomers (Fig. 3c) whether expressed as particle concentrations (bottom x-axis in graph 3c) or initial monomer concentrations (top x-axis in graph 3c). The unambiguous results we report here are in agreement with our previous observation that fibrillar α-syn is several orders of magnitude more toxic than a heterogenous mixture of oligomeric species (dimers, tetramers, higher order oligomers)^7^.

SDS-resistant α-syn oligomers obtained using DA and GA, made of ~20, 16, 6 and 4 monomers, respectively (Fig. 4), efficiently bound to the cell plasma membranes but did neither permeabilize them nor result in significant cytotoxicity (Fig. 5). DA-mediated α-syn oligomers have been considered relevant in the pathophysiology of PD, possibly accounting for the selective vulnerability of dopaminergic neurons in disease^57^. While DA has been shown to increase apoptosis in cultured neurons overexpressing α-syn^58^,^59^, other studies reported that increased DA levels favor the formation of intracellular innocuous soluble α-syn oligomers^31^,^60^ with a concomitant reduction in the amount of insoluble α-syn aggregates^31^. Our observation that extracellularly added α-syn oligomers, whether formed in the presence of DA or not, have similar low toxicities suggests that DA-mediated α-syn oligomers are not directly responsible for the selective vulnerability of dopaminergic neurons in PD.

Interestingly, large DA-mediated and large GA-cross-linked α-syn oligomers composed of ~20 and 16 monomers, respectively, seeded the aggregation of soluble α-syn in the cytoplasm of reporter neuroblastoma cells (Figs 6 and 7). Smaller oligomers did not. Altogether our findings suggest that low-molecular weight and unstable oligomers do not seed soluble α-syn assembly into fibrils, while high molecular weight and stable oligomers do although to a much lower extent than α-syn fibrils. Interestingly, AUC measurements indicate that the stable high molecular weight α-syn assemblies are elongated in shape. This strongly suggests that α-syn oligomers are a continuum of species ranging from unstable low molecular weight particles to stable short fibrils with seeding capacity, in other words that α-syn assembly follows an isodesmic, indefinite self-association mechanism. Further structural characterization of low and high-molecular weight α-syn species will allow defining how they relate to fibrils.

## Methods

### Expression and purification of α-syn

The *Escherichia coli* strain BL21(DE3) (Stratagene, La Jolla, CA, USA) was transformed with the expression vector pET3a encoding for human wild-type α-syn. α-syn was purified as described^61^. Protein concentration was determined spectrophotometrically using an extinction coefficient of 5960 M^−1^cm^−1^ at 280 nm. Pure α-syn (0.8–1 mM) in assembly buffer (50 mM Tris-HCl, pH 7.5, 150 mM KCl) was filtered through sterile 0.22-μm filters and stored at −80 °C.

### Assembly of monomeric α-syn into oligomers and fibrils

For spontaneous formation of on-fibrillar assembly pathway α-syn oligomers, α-syn was incubated at 800 μM in assembly buffer at 4 °C, without shaking, for 7 days.

For DA-mediated oligomerization, α-syn was incubated in assembly buffer in the presence of DA (Sigma) (molar ratio α-syn:DA 1:10) for 3 days at 37 °C without shaking.

For cross-linking with GA, α-syn (800 μM) was dialyzed against PBS pH 7.4 for 6 h at 4 °C and incubated in the presence of GA (Fluka) (molar ratio α-syn:GA 1:50) at room temperature for 30 min. The reaction was stopped by adding 50 mM Tris-HCl pH 7.5.

α-syn oligomeric species of different sizes were separated from the monomeric form of the protein by SEC on a Superose 6 HR10/300 column (GE Healthcare) equilibrated in PBS pH 7.4 diluted 10 folds at a flow rate of 0.5 ml/min. Elution was monitored by measuring absorbance at 280 nm. The Superose 6 column was calibrated with Dextran blue (over 2200 kDa), (670 kDa), β-amylase (200 kDa), BSA (66 kDa), and carbonic anhydrase (29 kDa) standards (Sigma).

The concentration of SEC-purified on-fibrillar assembly pathway α-syn oligomers was determined spectrophotometrically as described above. That of SEC-isolated DA-induced and GA-cross-linked α-syn oligomers was determined by 10% Tris-Tricine-SDS-PAGE quantification after Coomassie-blue staining and comparison to standards corresponding to different amounts of the total oligomeric mixture prior to loading onto the Superose 6 column ran on the same gel. Images were processed and quantified using the software ImageJ. Briefly, the intensity of each band was integrated using ≪rectangle selection≫ tool with a fixed size. The background was measured on a neighbouring area and subtracted. The different SEC-purified monomeric and oligomeric α-syn fractions in PBS diluted 10 folds were flash-frozen, concentrated 10-fold by lyophilization in a Christ 2–18 CD rotational vacuum concentrator connected to an Alpha 1–2 LD Plus freeze dryer (Martin Christ Gefriertrocknungsanlagen GmbH, Germany) and stored at −80 °C until use.

For assembly into fibrils, α-syn was incubated in assembly buffer at 37 °C under continuous shaking for 4 days^7^. Before experiments with cultured cells, α-syn fibrils were pelleted at 16000 × g for 15 min and resuspended twice in PBS pH 7.4. To obtain fibrils homogeneous in size, fibrillar α-syn was sonicated for 20 min on ice in 2 ml Eppendorf tubes in a VialTweeter powered by an ultrasonic processor UIS250v (250 watts, 24 kHz, Hielscher Ultrasonic, Teltow, Germany) set at 75% amplitude, 0.5 s pulses every 1 s. The morphology of α-syn assemblies was assessed by TEM in a Jeol 1400 transmission electron microscope following adsorption onto carbon-coated 200 mesh grids and negative staining with 1% uranyl acetate. The images were recorded with a Gatan Orius CCD camera (Gatan).

### Analytical ultracentrifugation

Sedimentation velocity measurements were carried out using a Beckman Optima XL-A ultracentrifuge equipped with UV-visible detection system using an AN60-Ti four-hole rotor and cells with two-channel 12 mm pathlength centerpieces. For monomeric and oligomeric α-syn, samples (400 μl in PBS pH 7.4 diluted 10 folds) were spun at 50000 rpm (182000 × *g*) and 25000 rpm (45500 × g), respectively, at 15 °C. For fibrillar α-syn the sample (400 μl in assembly buffer) was spun at 2000 rpm (290 × *g*) at 20 °C. Sample displacement profiles were obtained by recording the absorbance at 280 nm every 5 min. Sedimentation coefficient continuous c(s) and ls-g*(s) distributions were determined using the software Sedfit (National Institutes of Health, Bethesda, MD)^36^,^37^. The partial specific volume (0.7305 ml/g for monomeric and oligomeric α-syn, 0.7326 ml/g for fibrillar α-syn), the buffer viscosity (1.1386 cP for monomeric and oligomeric α-syn, 1.015 cP for fibrillar α-syn), and the buffer density (0.99913 g/ml for monomeric and oligomeric α-syn, 1.00674 g/ml for fibrillar α-syn) were calculated with the software Sednterp. For all the measurements, the sedimentation coefficient values were corrected to *s*_20,*w*_ (standard solvent conditions in water at 20 °C).

### α-syn labeling with extrinsic fluorophores, intracellular Ca^2+^ levels measurements and epifluorescence microscopy imaging

α-syn assemblies in PBS, pH 7.4 were labeled by addition of 2 molar excess of the aminoreactive fluorescent dyes ATTO488 or 550 (ATTO-Tech GmbH) following the manufacturer’s instructions. Labeled monomeric and oligomeric α-syn were separated from unreacted fluorophore by gel filtration on PD-10 desalting columns equilibrated in PBS pH 7.4 (GE Healthcare), while fibrillar α-syn was pelleted at 16000 × g for 15 min and resuspended twice in PBS pH 7.4.

Human neuroblastoma SH-SY5Y cells (ECACC; Sigma Aldrich, St. Louis, MO, USA) were cultured at 37 °C in humidified air with 5% CO_2_ Dulbecco’s modified Eagle’s Medium/Ham’s nutrient mixture F-12 (DMEM/F12) containing 10% fetal bovine serum, 2 mM glutamine, 100 units/ml penicillin, and 100 μg/ml streptomycin. All materials used for cell culture were from PAA Laboratoires GmbH (Pasching, Austria) unless otherwise stated.

SH-SY5Y cells cultured on ibidi-μ-Dishes (ibidi, Martinsried, Germany) were incubated for 15 min with ATTO550- or ATTO488-labeled α-syn assemblies (1 μM equivalent monomer concentration). Then, the cells were washed and immediately imaged in serum-free, phenol red-free DMEM/F12 on a Zeiss Axio Observer Z1 epifluorescence microscope equipped with an Incubator XLmulti S1 RED LS (Carl Zeiss) and an Orca-R^2^ camera (Hamamatsu). Immediately after imaging, 0.1% Trypan Blue (Sigma-Aldrich) was added to quench the fluorescence of ATTO-488-labeled α-syn assemblies that were plasma membrane-bound and not yet within cells. Cells were re-imaged at the same exposure time of the camera (300 ms) after the addition of Trypan Blue (dead time 30–60 s) to assess the amount of internalized α-syn assemblies.

To monitor intracellular free Ca^2+^ levels, subconfluent SH-SY5Y cells were loaded for 30 min with 5 μM of the cell permeable fluorescent Ca^2+^ indicator Fluo-4-AM (Invitrogen) in the presence of 0.02% (w/v) Pluronic Acid F127, a dispersing agent that facilitates Fluo-4-AM uptake by the cells, and 2.5 mM of the anion-transport inhibitor probenecid (Invitrogen) to reduce dye leakage. Before starting measurements, cells were washed and incubated for 30 min in serum-free, phenol red-free DMEM/F12 to allow complete de-esterification of intracellular Fluo-4-AM to Fluo-4. After addition of α-syn assemblies, cells were imaged on the Zeiss Axio Observer Z1 epifluorescence microscope every 30 s for 20 min at 37 °C in humidified air with 5% CO_2_ using the software Axiovision (Carl Zeiss). The fluorescence variation of at least 100 cellular areas from at least 3 independent experiments was quantified using the software ImageJ, after background subtraction for each image. The percentage of calcium influx was expressed as a fraction of the maximum fluorescence recorded after addition of the ionophore ionomycin (10 μM) (Invitrogen) at the end of each experiment.

### Cell viability assay

The toxicity of α-syn assemblies was assessed by the MTT reduction inhibition assay, a widely used indicator of cell viability, based on the ability of healthy cells to reduce the tetrazolium salt MTT to purple MTT formazan^62^,^63^. The assay was performed as previously described^7^. Briefly, SH-SY5Y cells were plated on 96-well plates at a density of 8000 cells per well. After two days, cells were exposed to increasing concentrations of the different α-syn assemblies (100 μl per well in fresh culture medium) for 24 h. After incubation, the medium containing α-syn assemblies was replaced by 100 μl of phenol red-free DMEM/F12 containing 0.5 mg/ml MTT (Sigma). After 4 h, cells were lysed by addition of 100 μl lysis solution (20% SDS, 50% *N*,*N-*dimethylformamide) and further incubated at 37 °C to allow complete sample homogeneization. The absorbance of the formazan was measured at 570 nm in a FlexStation3 microplate reader (Molecular Devices, Sunnyvale, CA).

### Seeding properties of α-syn assemblies

We used murine neuroblastoma Neuro2A cells (ATCC, Manassas, VA) stably expressing ChFP–α-syn^13^ to assess the ability of exogenously added α-syn assemblies to seed the aggregation of otherwise soluble endogenous cytoplasmic α-syn. Cells cultured on ibidi-μ-Dishes (ibidi, Martinsried, Germany) in DMEM containing 10% fetal bovine serum, 2 mM glutamine, 100 units/ml penicillin, 100 μg/ml streptomycin and 400 μg/mL neomycin (G418), were exposed to different concentrations of ATTO488-labeled α-syn assemblies. After 24 h, cells were washed and imaged in the Zeiss Axio Observer Z1 epifluorescence microscope at a 20× magnification. The percentage of cells with cytoplasmic ChFP/ATTO488 overlapping puncta was estimated by randomly counting at least 500 cells in 7–10 fields from three independent experiments.

### Proteinase K digestion of cell extracts and western blot analysis

Neuro2A cells stably expressing ChFP–α-syn grown on 60 cm^2^ Petri dishes were exposed to monomeric α-syn, fibrillar α-syn or large GA-cross-linked α-syn oligomers (5 μM equivalent monomer concentration). After 24 h, cells were washed twice, recovered in PBS (2 × 10^6 ^cells/ml) and lysed by sonication (3 × 10 s at an output power of 5 W rms) using a Branson Sonifier cell disrupter, model 150. Aliquots of each lysate (40 μl) were incubated with increasing concentrations of Proteinase K (Roche) (0.002–1 μg/ml) for 20 min at 37 °C. The proteolytic reactions were stopped by addition of 1 mM PMSF and the samples were immediately denatured in Laemmli buffer for 5 min at 95 °C. The samples were analyzed on 12% Tris-Glycine SDS-PAGE and western blotting on nitrocellulose membranes. ChFP–α-syn was probed with mouse monoclonal anti-α-syn antibody (BD Biosciences Cat #610787) and imaged on a ChemiDoc^TM^ MP Imaging System (Biorad). The immunoreactivity of α-tubulin (mouse monoclonal antibody DM1A, Abcam Cat #ab7291) was used as a loading control.

### Semi-Denaturing Detergent-Agarose Gel Electrophoresis (SDD-AGE)

SDD-AGE was performed as previously described^64^. Briefly, samples of α-syn monomers, fibrils or DA-mediated oligomers were mixed with 4X sample buffer (2× Tris acetate-EDTA (TAE), 20% glycerol, 8% SDS, 0.05% bromophenol blue) and incubated for 10 min at room temperature. Samples were then analyzed on a 1.6% agarose gel in TAE buffer containing 0.1% SDS, blotted onto nitrocellulose membranes by capillary transfer in Tris-buffered saline buffer, and probed with the mouse monoclonal anti-α-syn antibody (BD Biosciences Cat #610787).

## Additional Information

**How to cite this article**: Pieri, L. *et al*. Structural and functional properties of prefibrillar α-synuclein oligomers. *Sci. Rep.* **6**, 24526; doi: 10.1038/srep24526 (2016).

## Supplementary Material

Supplementary Information
