# Supplementary material for: Structural and functional properties of prefibrillar α-synuclein oligomers
Source: Sci Rep. 2016 Apr 14;6:24526. doi: 10.1038/srep24526 (PMC4830946; doi:10.1038/srep24526)
Supplement: Supplementary Information [file srep24526-s1.pdf]

Structural and functional properties of prefibrillar α-synuclein oligomers

Laura Pieri, Karine Madiona, Ronald Melki

Paris-Saclay Institute of Neuroscience, Centre National de la Recherche Scientifique, Université Paris-Saclay, 91190 Gif-sur-Yvette, France

Address correspondence to R. Melki, ronald.melki@cnrs.fr; Tel. +33169823503; Fax. +33169823447

SUPPLEMENTARY FIGURES

Supplementary figure S1.

TEM images of SEC-purified on-assembly pathway α-syn oligomers at different stages of incubation in PBS pH 7.4 at 37 °C under agitation. On-fibrillar assembly pathway α-syn oligomers are stable in morphology and size when incubated for up to 7 days at low concentration. Upon incubation at high concentrations (8 μM i.e. 250 μM equivalent monomer concentration), on-fibrillar assembly pathway α-syn oligomers give rise to chains and rings, concomitant with the appearance of short fibrils. At later incubation stages, more and more α-syn fibrils can be observed concomitant with a decrease in the amount of early oligomers. Scale bars represent 200 nm.

Supplementary figure S2.

Coomassie blue stained 10% Tris-Tricine SDS-PAGE of the different DA-induced (**a**) and GA-cross-linked α-syn oligomers (**b**) separated by SEC (see Fig. 4). Elution fractions of interest (0.5 ml each) indicated by frames were pooled and their structural and functional properties were assessed.

Supplementary figure S3.

(**a**) SDD-AGE and immunoblot analysis of DA-mediated α-syn oligomers before and after 24 h incubation at 1.25 μM (i.e. 25 μM equivalent monomer concentration) at 37 °C in serum free DMEM/F12 cell culture medium. The SDD-AGE profile of DA-mediated α-syn oligomers after incubation was identical to that of freshly purified DA-mediated α-syn oligomers, but different from that of monomeric and fibrillar α-syn. 2.5 μg α-syn were loaded in each lane, and the blot was probed with mouse monoclonal anti-α-syn antibody (BD Biosciences, Cat # 610787). (**b**) TEM images of DA-mediated α-syn oligomers before and after incubation at 25 μM (i.e. 500 μM equivalent monomer concentration) for 7 days at 37 °C. Scale bars represent 200 nm.

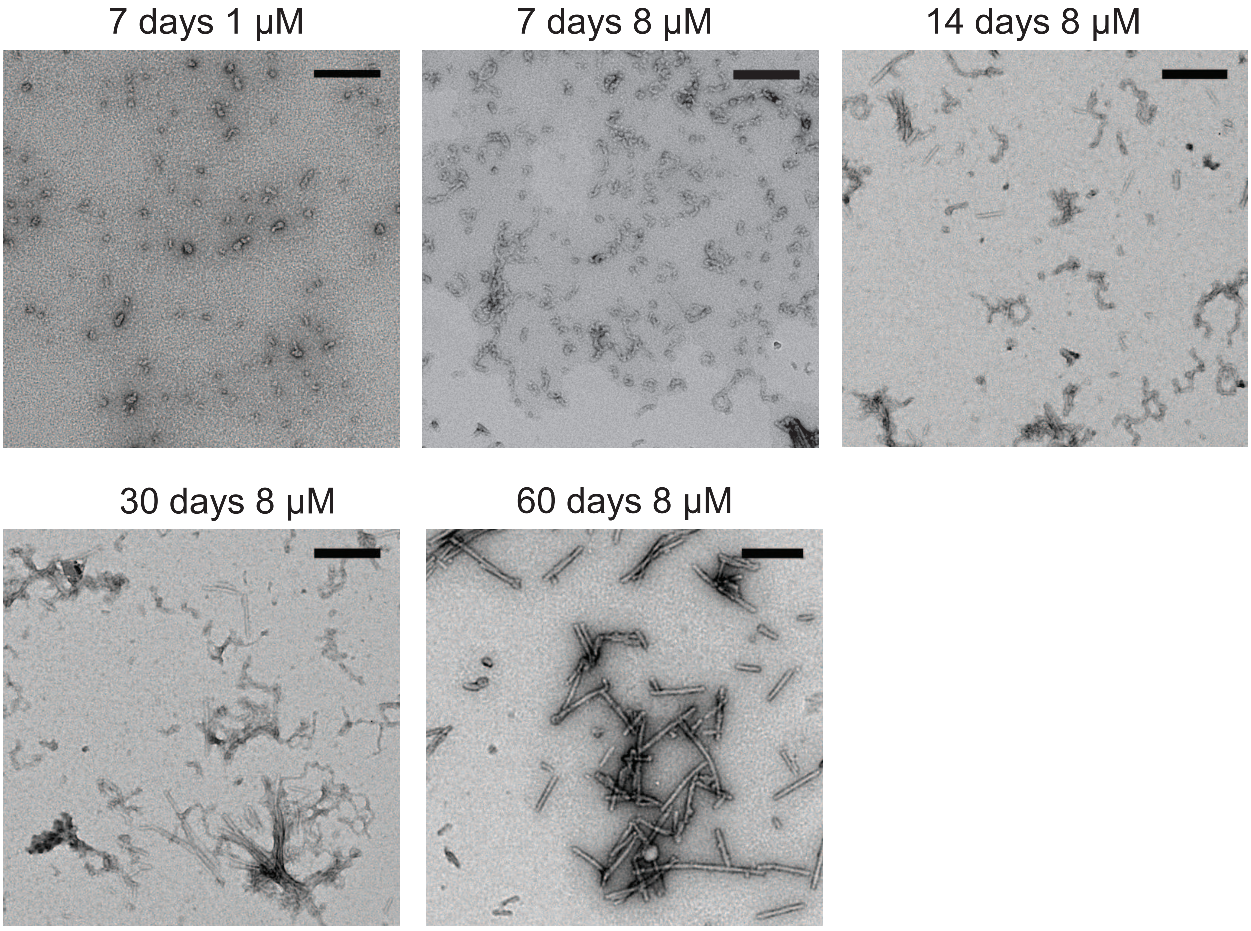

Supplementary figure S1

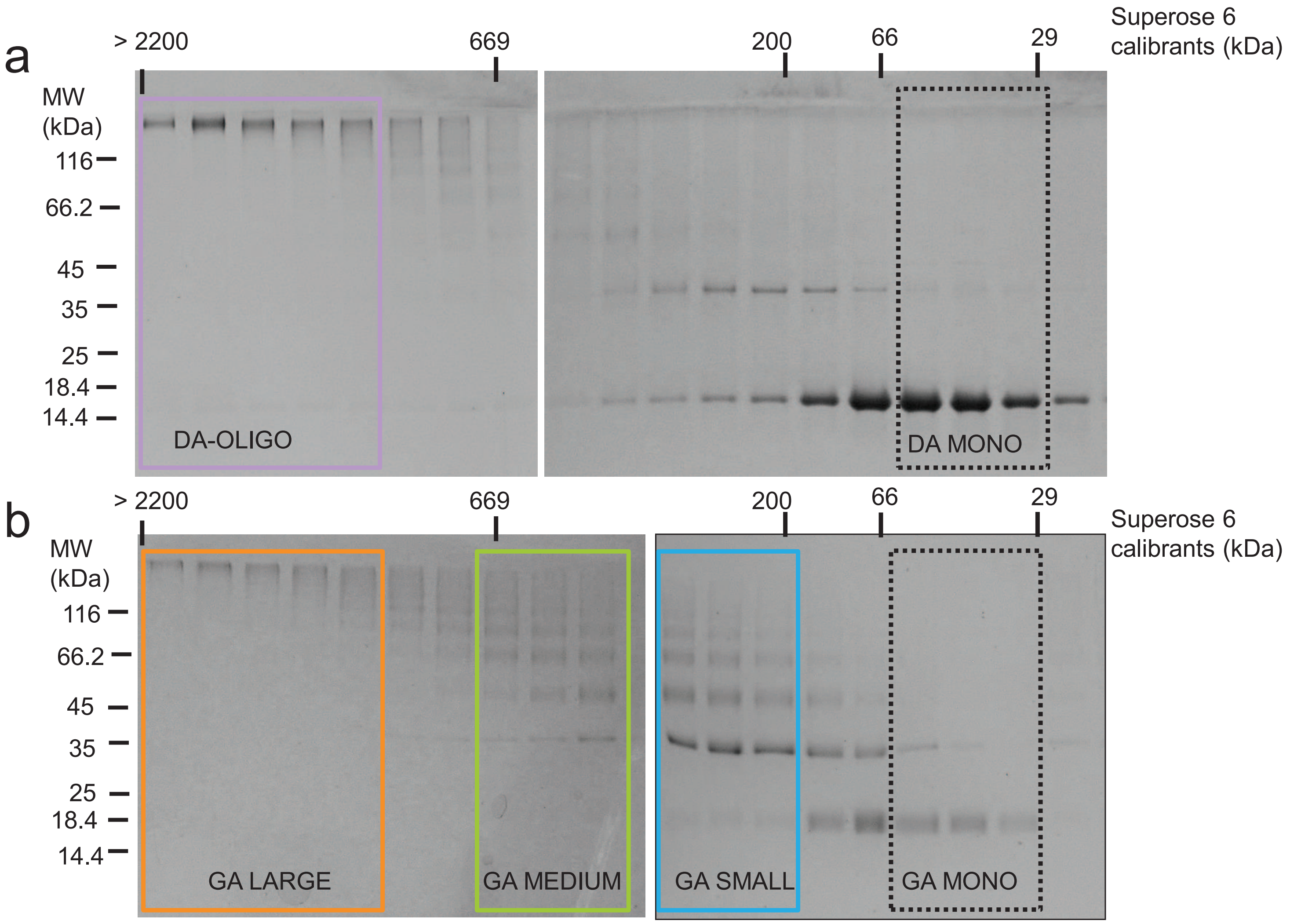

Supplementary figure S2

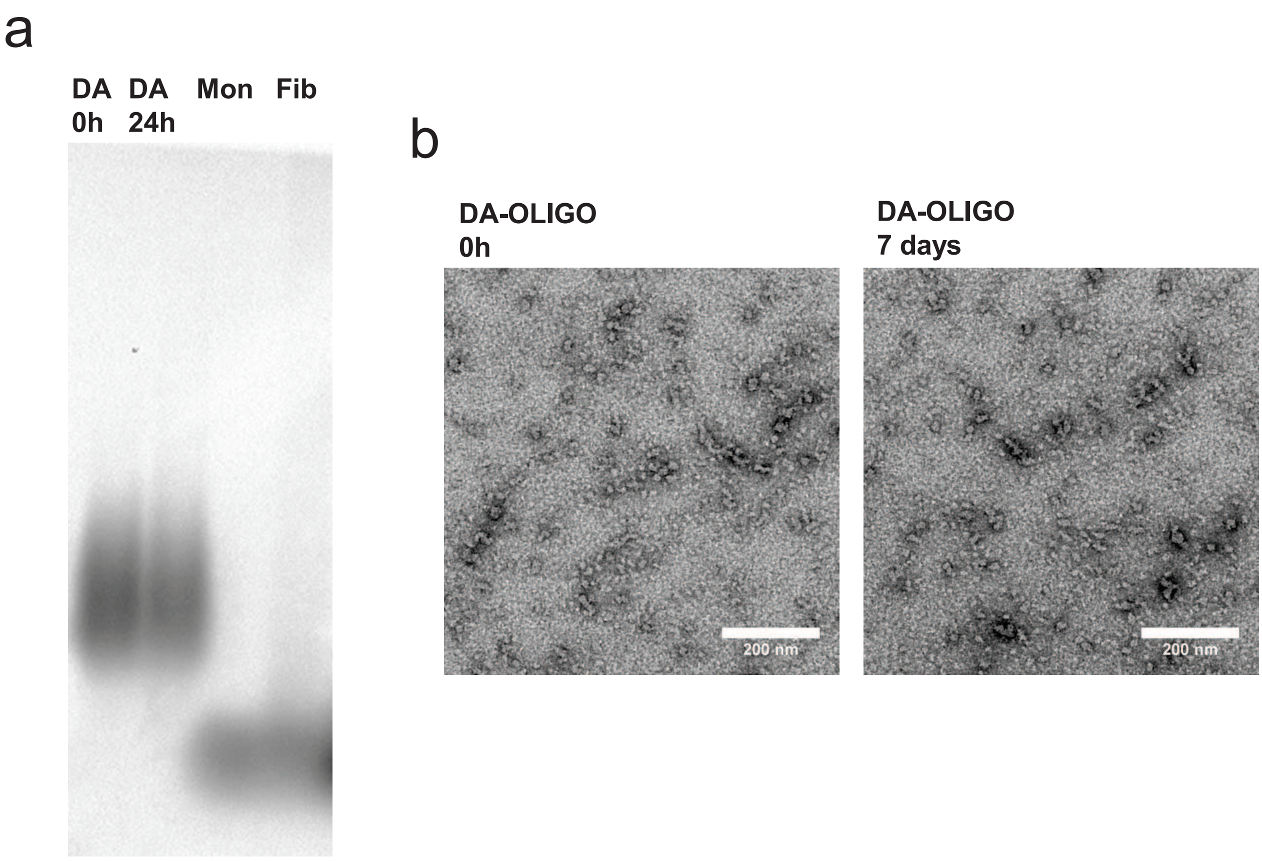

Supplementary figure S3
